# The Molecular and Phenotypic Basis of the Glioma Invasive Perivascular Niche

**DOI:** 10.3390/ijms18112342

**Published:** 2017-11-06

**Authors:** Mohammed Diksin, Stuart J. Smith, Ruman Rahman

**Affiliations:** Children’s Brain Tumour Research Centre, School of Medicine, University of Nottingham, Nottingham NG7 2UH, UK; mohammed.diksin@nottingham.ac.uk (M.D.); stuart.smith@nottingham.ac.uk (S.J.M.)

**Keywords:** glioblastoma, tumour invasion, perivascular niche, extracellular matrix, chemokine

## Abstract

Gliomas are devastating brain cancers that have poor prognostic outcomes for their patients. Short overall patient survival is due to a lack of durable, efficacious treatment options. Such therapeutic difficulties exist, in part, due to several glioma survival adaptations and mechanisms, which allow glioma cells to repurpose paracrine signalling pathways and ion channels within discreet microenvironments. These Darwinian adaptations facilitate invasion into brain parenchyma and perivascular space or promote evasion from anti-cancer defence mechanisms. Ultimately, this culminates in glioma repopulation and migration at distances beyond the original tumour site, which is a considerable obstacle for effective treatment. After an era of failed phase II trials targeting individual signalling pathways, coupled to our increasing knowledge of glioma sub-clonal divergence, combinatorial therapeutic approaches which target multiple molecular pathways and mechanisms will be necessary for better treatment outcomes in treating malignant gliomas. Furthermore, next-generation therapy which focuses on infiltrative tumour phenotypes and disruption of the vascular and perivascular microenvironments harbouring residual disease cells offers optimism for the localised control of malignant gliomas.

## 1. Introduction

High-grade gliomas remain one of the most aggressive and difficult to treat cancers in adults, with a very poor prognosis of 14 months [1] despite current multimodal therapeutic approaches. Unlike other solid tumours, high grade gliomas rarely metastasise outside the brain via haematological and lymphatic vessels [2]. However, local invasiveness of these tumours through normal brain tissue is one of the main challenges for more efficacious treatment. Glioma cells which have infiltrated the surrounding parenchyma of the normal brain and along nearby blood vessels, cannot be safely surgically resected. Moreover, the intra-tumour genetic heterogeneity emerging from the dynamism of clonal selection processes within spatially distinct niches allow glioma cells to escape conventional chemotherapeutic and radiological treatments. Furthermore, temozolomide (standard-of-care chemotherapy) may confer a stringent selection pressure, by which the acquisition of mutations in the protein kinase B/mammalian target of rapamycin (AKT/mTOR), epidermal growth factor receptor (EGFR), platelet-derived growth factor receptor (PDGFR), c-Jun N-terminal kinase-extracellular signal-regulated kinase ½ (JNK-ERK1/2) and retinoblastoma pathways, may facilitate disease recurrence [3,4]. Similarly, radiotherapy was shown to enrich for stemness genotypes and phenotypes in some tumour subpopulations which might initiate tumour resistance and propagate disease progression [5]. This stem-like property in tumour subpopulations, which is intrinsic to cell survival or selected for during aggressive treatment approaches, is another confounding factor in tumour therapy because of the self-renewal capacities of glioma stem cells (GSC). This sub-population can initiate tumour formation, enhance tumour progression [6] and can be influenced by distinct tumour micro-compartments, where some GSC may ultimately differentiate into daughter cells of different lineages with distinct genetic, and epigenetic marks. The lack of any static and definitive markers of GSC to discriminate them from the normal neural stem cell counterparts is another confounding factor [7]. Moreover, the astrocytic marker, glial fibrillary acidic protein (GFAP), cannot fully discriminate between glioma progenitors and further differentiated glioma cells. Historically, glioma studies have utilised clinical samples surgically resected from tumour core regions; in hindsight, a reductionist approach which ignores the intra-tumour heterogeneity of genetic and epigenetic profiles observed within spatially distinct regions [8,9]. Glioma core regions are usually necrotic due to a rapidly growing tumour mass and inter-cellular competition for oxygen and nutrients; tumour edges represent a key hallmark of gliomas that is more clinically-relevant when considering next-generation therapy. Glioma invasive peripheries form a characteristic perivascular and perineural satellitosis. Ignoring these infiltrative tumour peripheries obscures the full biological profile of the invasive process, where residual infiltrating tumour cells left after maximum surgical removal, ultimately give rise to malignant glioma recurrence.

Glioma aggressiveness and therapeutic resistance has led to many transcriptome- or genome-wide studies aiming to unravel the complex molecular pathways of gliomas to better understand disease progression and to design novel targeted therapeutic agents. For instance, efforts have been made to research and analyse thousands of genetic profiles of tumour samples uploaded in a shared online space called The Cancer Genome Atlas Research Network in order to identify appropriate tumour targets [10,11,12]. Additionally, elegant studies involving multi-region sampling were conducted to decipher the co-relation between the genetic [3] and epigenetic [4] profiles of high-grade glioma recurrences and their patient-matched low-grade gliomas. Although distinct genetic and epigenetic growth drivers have been observed, no clinical impact has been achieved so far by purported targeted therapies. In this review, we aim to give some insights into the perivascular compartment during glioma cell invasion, which is important for a better understanding of tumour progression, and ultimately developing new translational research trends and prioritisation of novel therapeutic agents.

## 2. The Glioma Invasive Phenotype

The invasive ability of gliomas has been the focus of pathologists since 1938, when German neuropathologist Hans Joachim Scherer suggested that the tumour cells of glioma malignancies infiltrate the normal brain parenchyma with distinct morphological patterns [13]. Of these, he categorised the secondary structures, in which Scherer described the arrangement of the invading glioma cells in relation to neural and glial cells or alongside the white matter tracts, blood vessels and meninges. In particular, he described the perivascular satellitosis; i.e., the concentric arrangement of glioma cells outside the Virchow–Robin spaces of the pre-existing normal brain vessels. Scherer also described the predilection of the invasive malignant glioma cells to the capillaries and small vessels as one of the distinctive features of glioma tumours from the perivascular gliosis. In addition, he observed that glioma cells grow around blood vessels in areas of normal parenchymal brain tissue at some distance from the original tumour mass. This view of the invasiveness of gliomas is still valid and accepted [14,15,16] and has been further corroborated by several recent studies. For example, the attachment of migrating glioma cells depends on receptors expressed on the surface of cells and on the extracellular matrix and is mediated through cell-cell and cell-matrix interaction by adhesion molecules such as integrins and cadherins. The detachment of these invading cells occurs mainly through the degradation and remodelling of the surrounding extracellular matrix by matrix metalloproteinases [17,18,19,20,21]. According to this hypothesis, malignant glioma cells detach from their primary tumour, create new connections with surrounding parenchyma, destroy and remodel the extracellular matrix and finally migrate into healthy tissue [22]. Similarly, some studies found that glioma cells undergo several complex morphological changes, which are mediated by the actin-myosin machinery, to enable migration into surrounding brain tissue [23,24]. Additionally, these significant volume and shape changes of glioma cells during the invasion process were observed by in vivo and ex vivo time-lapse imaging techniques [14,25]. For example, significant hydrodynamic glioma cell volume changes (that might reach up to 33% reduction of glioma cell volume) could occur to facilitate invasion through small-sized surrounding spaces. This was inferred from in vitro observations where a one-third reduction in glioma cell volume preceded infiltration through transwell barriers with 3–8 μm pore size [25]. These periodic morphological changes were achieved by repurposing ion-channels that are normally used to control normal neural excitability functions [26]. Such complex sequential mechanisms allow the invasion of glioma cells into narrow compartments such as the perivascular space which otherwise would be too small for the cells [26].

Among all spaces provided by the Scherer model of invasion, the perivascular space has the major share of glioma invasion, and more than 85% of the invading malignant cells migrate around blood vessels to form the satellite tumour shape [16,27]. Furthermore, it could be argued that glioma cells invade the vascular compartment and co-opt pre-existing vessels [27] early during disease progression without the need for the neo-angiogenic factors produced by glioma tumour cells. This may explain the failure of all anti-angiogenic drugs tested in clinical trials as a first line treatment option. For example, bevacizumab has failed to show any overall survival benefit in newly diagnosed glioma patients [28]. Moreover, at least some glioma cells retain or acquire the ability to create tumour-derived vascular networks toward late-stages of the disease in a phenomenon termed vascular mimicry [29,30]. El Hallani and colleagues showed that non-endothelial cells derived from glioblastoma multiforme (GBM), which exhibited stem-like features, could secrete pro-angiogenic factors and express endothelial markers, mimicking features of vascular smooth muscle-like cells. Vascularity in gliomas was shown to be generated in both an oxygen-dependent and oxygen-independent manner via the master angiogenic regulators vascular endothelial growth factor (VEGF) and hypoxia induced factor-2α (HIF-2α) or through fibroblast growth factor 1 (FGF1) signalling pathways respectively [31,32,33].

## 3. Molecular Basis of Glioma Perivascular Invasion

**Figure 1:**
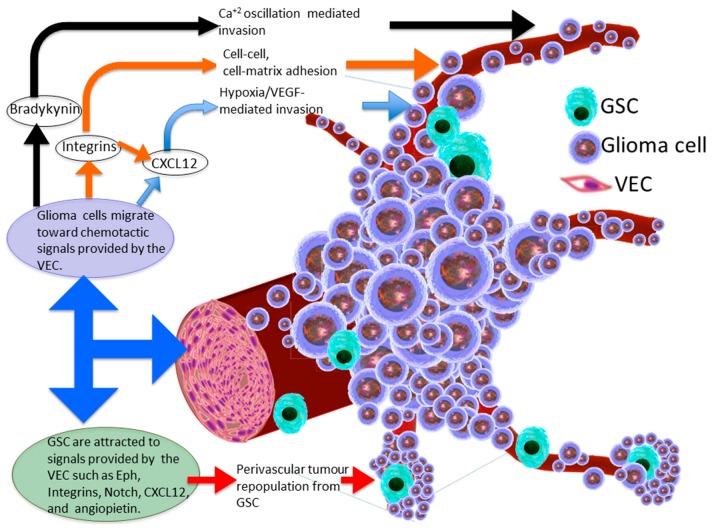
The glioma invasive perivascular niche. Characteristic perivascular satellitosis occurs due to the migration of glioma cells towards ligands expressed by the vascular endothelial cells (VEC). These chemotactic molecules are also capable of attracting glioma stem cells (GSC) towards the perivascular niche via several molecular pathways. Bradykinin is an inflammatory mediator causing blood vessels to dilate, promoting the chemotactic invasion of malignant gliomas. Vascular endothelial growth factor (VEGF); chemokine (C-X-C motif) ligand 12 (CXCL12).

Despite a predilection in gliomas to vascularity, it is not clear yet whether glioma cells infiltrate this perivascular space preferentially as a response to appropriate nutrients and environmental cues which promote tumour survival and growth, or whether this space represents the least physical barrier that resists tumour cell propagation to surroundings. According to our current knowledge, this question is yet to be answered and therefore needs to be comprehensively addressed, as it affects the trends and the prioritisation of tumour treatment modalities. It could be argued that the availability of certain chemo-attractive molecules in the perivascular and endothelial cell niches could explain the preferential habitation of glioma cells around the vascular tree (Figure 1). For example, Bradykinin (BK) provides chemotactic signalling to glioma cells in the perivascular niche [16]. BK cleavage from high molecular weight kininogen is initiated by vascular endothelial cells (VEC) via the activation of the kallikrein–kinin system, which ultimately leads to the conversion of pre-kallikrein into kallikrein [34]. Kallikreins are a subgroup of serine proteases which coordinate various physiological functions including blood pressure. Binding of BK to its receptors leads to the activation of G protein-coupled receptors which increases Ca^2+^ concentrations through inositol-1,4,5-triphosphate receptor 3 [35,36]. Calcium concentrations under the regulation of BK levels were shown to be crucial to glioma invasion [16]; i.e., low BK levels caused a prolonged persistence of intracellular calcium [16,37], whereas long exposure to BK lead to Ca^2+^ oscillations [16,38]. These incremental alterations in the intracellular Ca^2+^ resulted in the activation of ion channels necessary for volume and morphology changes of glioma cells during migration through narrow spaces. Calcium oscillations results in reprogramming of the Cl^−^ and K^+^ channels which are normally set to regulate neural membrane potential. This enables the glioma cell to reduce its volume down to 33% of its original size, and thus facilitating the infiltration through small compartments by removing free cytoplasmic water outside the cell via Cl^−^ efflux [26,39]. Seifert and Sontheimer (2014) showed that BK may enhance amoeboid glioma cell migration via mobilisation of the intracellular Ca^2+^ which, in turn, induces the contraction of cellular cytoskeleton, cytosolic flow and ultimately the formation of bleb protrusions at glioma cell membranes. Both BK receptor 2 inhibitor, Hoe-140, and bleb retraction blocker, blebbistatin, were both effective in inhibiting glioma cell invasion [40]. Montana and Sontheimer (2011) further suggested that not only is BK a key ligand for glioma cell invasion via fluctuation of Ca^2+^ levels, but it can also enhance human and rat glioma cell migration in vitro via binding to BK receptor 1. This leads to the activation of the phosphatidylinositol-4,5-bisphosphate 3-kinase (PI3K)/AKT cascade signalling pathway [41], involved in cellular growth and metabolism [42], or the release of several molecules from astrocytes such as D-serine, ATP, and glutamate which in turn support and stimulate glioma invasion [43,44,45,46,47]. It is noteworthy to observe that in addition to the major role of BK in glioma cell migration around the perivascular space, it can promote the migration of glioma cells into the surrounding brain matrix by remodelling this compartment via matrix metalloproteinase [48].

As a consequence of the significant role of BK in glioma perivascular satellitosis, a BK receptor inhibitor, Icatibant (Firazyr; Shire), has been tested in preclinical studies using glioma rat models and shown to be effective in impairing the migration of glioma cells through cerebral parenchyma and ultimately resulting in a smaller tumour mass [16]. Similarly, a family of cell surface integrin receptors (e.g., integrin β subunit when heterodimerised with the α-subunit), attach to collagen, laminin, fibronectin, vitronectin, osteopontin and tenascins of the perivascular extra cellular matrix and have been observed to be important in glioma invasion [19]. Thus, these integrins have been targeted by many pharmacological blockers proposed in preclinical studies and been tested in clinical trials. Despite the strong relationship between integrins and the glioma cell invasion phenotype, no clinical impact has been demonstrated in clinical trials. For example, cilengitide which showed moderate efficacy in Phase II trials, has failed to add any overall survival benefit in Phase III trials [49].

## 4. Chemokine Signalling within the Perivascular Invasive Niche

Integrins likely also promote perivascular invasion through the activation of the chemokine (C-X-C motif) ligand 12 (CXCL12)/CXCR4) signalling pathway [50]. CXCL12, previously known as induced stromal cell-derived factor-1α, is a key signalling molecule, hypothesised to act through CXCR4/CXCR7 receptors as a chemotactic ligand for glioma cells to invade the perivascular compartment [15,51]. Zagzag and colleagues observed that CXCL12 induced a strong transcriptomic signal in neurons and vessels bordering with the invading edge of mouse brain gliomas. CXCL12 was upregulated in normoxic conditions in vitro after paracrine exposure of neuronal and endothelial cells to VEGF secreted by the nearby perivascular fibroblasts and by tumour cells in an autocrine manner. Additionally, CXCL12 was shown to selectively attract and positively stimulate the migration of glioma cells which exclusively upregulated the expression of CXCR4 receptors [15]. Alternatively, hypoxia, which usually develops in the tumour core region progressively with the growing tumour mass, leads to an increase in CXCR7 expression in the microvascular endothelium and ultimately enhances CXCL12-dependant glioma cell migration [52,53]. This was also corroborated by Liu and colleagues who suggested that glioma cells which express CXCR7 were shown to migrate toward CXCL12 gradients in close vicinity to blood vessels of highly vascularised glioma tumours [51]. Another in vitro study conducted by Yadav and colleagues showed that human and murine GSC can migrate toward brain VEC via activation of the CXCL12/CXCR4 pathway, and CXCR4 genetic knockdown in a mouse model or pharmacological block using small molecule inhibitor AMD3100 (Plerixafor) leads to the reduction of tumour growth and vascular invasion [54]. There are two currently open clinical trials involving Plerixafor treatment in newly diagnosed patients with high-grade glioma (clinical trials.gov). The CXCL12–CXCR4/CXCR7 pathway is fully reviewed in [50]. Recently, it was shown that CXCR4 GSC are attracted to the perivascular space and transforming growth factor beta (TGF-β) expressed by endothelial cells canguide the differentiation of CXCR4 expressing GSC into mature pericytes to support tumour vascular sprouting and further growth [55].

## 5. Perivascular Niche Enhances Glioma Stem Cell Invasion

The GSC theory postulates an explanation for tumour aetiology, albeit the dynamic nature of GSC subpopulations cautions against reductionist descriptions of static populations. It suggests that the bulk of genotypically diverse tumour could be generated by a small population of self-renewing cancer stem-like cells that in turn can differentiate into multiple tumour cell lineages [6]. However, as is the case for any solid tissue cancer stem cells, it is yet to be identified whether these GSC arise from normal brain stem cells or from reprogramming of glial progenitors or mature differentiated cells. It is likely that many or all cells within glioma tumours demonstrate some degree of stem-like phenotype and that this cellular characteristic is likely to vary depending on micro-environmental conditions. In 2007, Calabrese and colleagues described the predilection of GSC (glioma cells expressing the neural stem/progenitor markers Nestin and CD133) to the endothelium of blood vessels and suggested that factors such as pigment epithelium-derived factor, and stem cell factor in the perivascular microenvironment are responsible for maintaining the self-renewal and proliferation potential of this population of cells [56]. Another study showed that Nestin^+^/CD133^+^ GSCs are located around CD31^+^ endothelial cells [57]; likewise, Yadav and colleagues (2016) showed the migration capacity of GSC toward brain VEC through activation of the CXCL12/CXCR4 pathway and where blocking CXCR4 signalling inhibits the invasive phenotype of GSC, rendering them more vulnerable and sensitive to radiotherapy [54]. Recently, it was shown that ephrin-B2 expressed in GSC may also play a significant role in perivascular invasion of GSCs by two mechanisms; firstly, Eph activation leads to the expulsion of these GSC within the tumour mass, which ultimately potentiates GSC motility and results in dissemination of individual cells away from the original tumour; secondly, Eph activation leads to the repression of sensitisation of these escaped GSC to the surrounding VEC-derived ephrin-B2, and thus “hijacks” the signalling pathway by which the normal vasculature inhibits the formation of tumour [7]. In addition, knock down of the gene encoding for ephrins (EFNB2) in GSC derived from patients’ samples or treating tumours generated from these GSC with anti-ephrin-B2 antibodies, showed a significant reduction in the initiation and progression of glioma tumourigenesis.

## 6. Vascular Endothelial Cell-Glioma Stem Cell Cross-Talk

Despite the anti-tumour activity of endothelial cells advocated by studies conducted in other tumours such as colorectal and prostate cancers [58,59], several studies showed the remarkable role of VEC in providing the appropriate microenvironment for GSC invasion and survival via the secretion of several factors which can induce multiple signalling pathways [56]. Of these, immunofluorescent staining of GSC in GBM revealed the upregulation of Notch receptors 1 and 2 in a region with close approximation to VEC expressing Notch ligands JAGGED 1/2 (JAG1/2), and delta-like ligand 4 (DLI4), which have been shown to be vital for the self-renewal capacity of GSC [60]. Moreover, nitric oxide provided by VEC was shown to switch on GSC self-renewal and to promote glioma tumorigenesis via Notch signalling [61]. Several other factors which are expressed by VEC and involved in GSC invasion and survival such as angiopoietin (via the activation of Tie2 receptor), leads to the expression of extracellular adhesion molecules such as N-cadherin and integrin β1, that may plausibly enhance GSC invasion [62]. Finally, VEC was shown to support GSC proliferation through activation of sonic hedgehog and mTOR signalling pathways [63,64].

## 7. Heterogeneous Location of Glioma Stem Cells

Current consensus in the glioma field suggests that GSC are located within two main compartments; firstly, GSC could be located where hypoxia is evident, and pseudopalisading glioma cells (a pathognomonic feature observed by light microscopy during histopathological diagnosis of GBM) are gathered with this characteristic form around the necrotic regions in the glioma core [65]; the second compartment is located around blood vessels that are present in the periphery of the tumour to form the perivascular satellitosis as discussed above. Most studies using patient-derived tissue usually involve a single specimen for each tumour per patient, which is usually taken surgically from the core region of the glioma. However, it is reasonable to speculate that the main difference between GSC in the core of the tumour and the periphery is that surgical intervention may remove the bulk of tumour core while the peripheral residual invasive GSC are left even after extensive surgical removal and ultimately contributing to disease recurrence and treatment resistance [66]. However, one cannot exclude the possibility that the combination of multi-modal therapeutic approaches confers a stringent selection pressure within distinct tumour micro-environments, thereby inducing subpopulations of glioma cells to initiate self-renewal programs that facilitate the repopulation of recurrent glioma. Thus, we advocate the importance of multi-region sampling in studies involving tumour specimens to better understand invasive GSC at the tumour edge, enabling the development of novel therapies targeting tumour invasion, recurrence, and treatment resistance.

## 8. The Gliovascular Regulatory Unit

**Figure 2:**
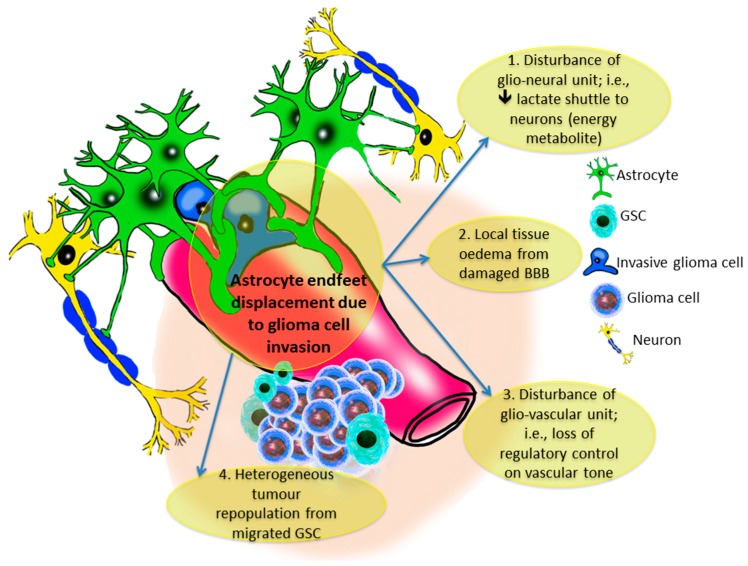
Astrocytic end-feet retraction due to the perivascular invasion of glioma tumour cells and/or glioma stem cells (GSCs). The function of both glio-neural and glio-vascular units are affected, leading to an increase in the neural excitotoxicity of neural tissue. Such toxicity includes local tissue oedema, vascular tone dysregulation and tumour repopulation from migratory GSC.

Current studies indicate that the infiltration of glioma cells through the perivascular space of the adjacent surrounding parenchyma has several dismal effects on the locally invaded niche of brain tissue. In normal physiological conditions, astrocytic end-feet circumferentially covers almost the entire surface of blood vessels [67] to form an interactive unit that is required for regulating neural function, maintaining the integrity of the blood-brain barrier (BBB), controlling vascular tone, and coordinating ion and metabolite exchange through specialised channels [68]. During glioma invasion through the perivascular space, the amoeboid processes of the migrating malignant cells elevate the end-feet of the astrocytes from the abluminal surface of blood vessels (Figure 2), which in turn disrupts and even breaches the adjacent BBB [27]. This disfigurement of the gliovascular regulatory unit results in the loss of control of normal astrocytes on the vascular tone via the Ca^2+^-dependant release of K^+^. Moreover, this also leads to several dysfunctional consequences on the delicate neurovascular unit such as the decrement of shuttling energetic metabolites such as lactate from astrocytes to neurons [69], and the reduction of blood flow previously observed in glioma patients [70]. The repurposing of vascular tone achieved by the migrating glioma cells was attributed to the tumour survival capability as vasoconstriction was hypothesised to be important for increasing the volume of the perivascular compartment during invasion, whereas vasodilatation is thought to be essential for the growing malignant mass [27]. The displacement of the astrocytic end-feet can also lead to disruption of the BBB and results in increasing vascular permeability, which in turn facilitates serum leakage into the surrounding region of brain tissue. However, it is not yet known whether these leaky vessels are due to the physical damage caused by the glioma cells while lifting the astrocytic end-feet from the blood vessel basement membrane, or due to down-regulation of the tight junction proteins (e.g., claudins) in the endothelial cells of the vasculature [27].

## 9. Conclusions

Although the definitive mechanism of glioma invasion through the perivascular space is largely unclear, we advocate that gliomas and GSC seek to invade the perivascular compartment to reach the abluminal surface of the blood vessels via tracking different chemotactic ligands, activating signalling pathways, expressing extracellular adhesive molecules, and even reprogramming the normal protective mechanisms towards tumour benefit. Such mechanisms hint at the adaptive nature of glioma cells as the perivascular compartment provides several advantages for the tumour, in addition to nutrients provided in the serum leaked trough fragile basement membranes of the invaded blood vessels. For instance, the generation of new vascular stem microenvironments by the migrating GSC could lead to considerable repopulation of tumour, with a completely distinct genetic and epigenetic profile and facilitate further tumour growth and invasion at the new site [56]. This niche also provides the appropriate environment for GSC to proliferate and invade via different molecular pathways. GSC can secrete VEGF which could lead to endothelial migration and ultimately promote neo-angiogenesis [56]. Furthermore, trans-differentiation of GBM stem-like cells into endothelial cells and/or vasculogenic mimicry could further contribute to tumour circulation [71]. As the vascular niche may provide a protective shield for GSC against chemo- and radiotherapies [56,72,73], targeting the dynamic GSC compartment of the invasive residual glioma is essential to inhibit tumour repopulation, differentiation into multiple cell lineages, migration and ultimately suppress the prolonged survival of tumour cells. We encourage a shift in philosophy from exclusively devising therapeutic strategies which target proliferation (i.e., canonical receptor kinases or cell cycle regulators) to including considerations for targeting the molecular basis of malignant glioma infiltrative phenotypes. Furthermore, impairment of glioma invasion may also enhance adjuvant cytotoxic chemo- and radiotherapy by potentially ensuring that residual disease remains local to the primary tumour site, rather than have penetrated deep into brain parenchyma.
